# Impaired consciousness due to injury of the ascending reticular activating system in a patient with bilateral pontine infarction: A case report

**DOI:** 10.1515/tnsci-2020-0138

**Published:** 2020-08-24

**Authors:** Soyoung Kwak, Min Cheol Chang

**Affiliations:** Department of Physical Medicine and Rehabilitation, College of Medicine, Yeungnam University, 170 Hyunchung-ro, Namgu, Daegu, 42415, Republic of Korea

**Keywords:** pontine infarction, ascending reticular activating system, consciousness, diffusion tensor tractography

## Abstract

The ascending reticular activating system (ARAS) is known to play an essential role in maintaining arousal and consciousness. In this report, we describe the case of a patient with impaired consciousness due to injury of the ARAS after bilateral pontine infarction. A 73-year-old female patient presented with anterior chest pain to the Emergency Department of our university hospital. She was diagnosed with chronic stable angina pectoris, three-vessel disease, and chronic total occlusion of the left anterior descending artery by coronary angiography and received conservative treatment. After five days, she showed deep drowsy mentality and brain MRI revealed bilateral paramedian pontine infarction. Four weeks after the pontine infarction, she showed severely impaired consciousness, with a Glasgow Coma Scale score of 7 (eye-opening: 2, best verbal response: 2, and best motor response: 3). Coma Recovery Scale-Revised score was 10 (auditory function: 2, visual function: 3, motor function: 2, verbal function: 2, communication: 0, and arousal: 1). Results of diffusion tensor tractography (DTT) for the ARAS showed decreased neural connectivity in the left lower dorsal ARAS, both lower ventral ARAS, and both upper ARAS. To the best of our knowledge, this is the first report of injury to the ARAS in bilateral pontine infarction diagnosed by DTT. We presume that our report would provide clinicians a better understanding of the mechanism of impaired consciousness in patients with pontine infarction.

## 1 Introduction

The ascending reticular activating system (ARAS) is known to play an essential role in maintaining arousal and consciousness [1,2]. The association between injury of the ARAS and impairment of consciousness has been reported in patients with various brain pathologies including stroke, traumatic brain injury, and hypoxic-ischemic brain injury [3]. The investigations for evaluation of the ARAS include positron emission tomography, electroencephalography, transcranial magnetic stimulation, and diffusion tensor tractography (DTT) [4,5,6]. Among these, DTT, derived from diffusion tensor imaging (DTI), has been suggested superior to other modalities. DTT enables three-dimensional visualization of white matter neural tracts and can reconstruct the ARAS in detail by segmentation (e.g. the lower dorsal ARAS, connecting the pontine reticular formation to the intralaminar thalamic nuclei; the lower ventral ARAS, connecting the pontine reticular formation to the hypothalamus; and the upper ARAS, connecting the intralaminar thalamic nuclei to the cerebral cortex) [7,8,9,10].

Impaired consciousness is one of the clinical symptoms that can occur following pontine infarct [11]. Pontine infarcts have been reported to account for 8% of ischemic stroke cases, which caused impaired consciousness [11]. Injury of the ARAS is known to cause impairment of consciousness [12]. Several previous studies have reported the association between injury of the ARAS and impaired consciousness in patients with pontine hemorrhage, intracerebral hemorrhage, traumatic brain injury, and hypoxic-ischemic brain injury using DTT [12,13,14,15,16,17]. However, there are limited studies evaluating the ARAS using DTT in patients with impaired consciousness following pontine infarction. In this report, we describe the case of a patient with impaired consciousness and injury of the ARAS after bilateral pontine infarction.

## 2 Patients and methods

### 2.1 Case report

**Figure 1:**
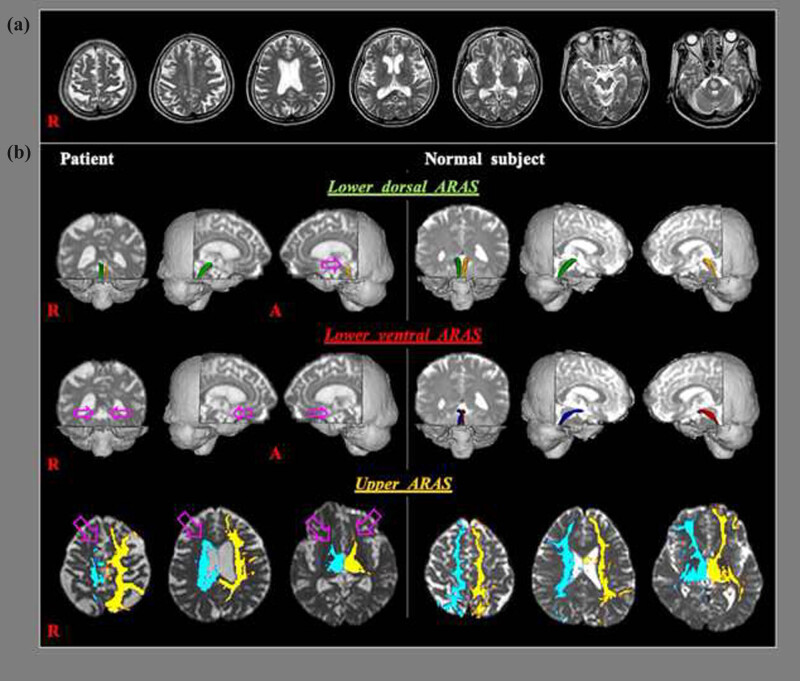
(a) Brain magnetic resonance images taken four weeks after the onset of pontine infarction showing bilateral leukomalatic lesion in the pons. (b) Results of DTT of the ARAS. Impaired neural connectivity (magenta arrows) in the left lower dorsal ARAS (the left lower dorsal ARAS [orange colored tract] is thinner), both lower ventral ARAS (the lower ventral ARAS was not reconstructed in both hemisphere) and both upper ARAS (decreased neural connectivity to each cerebral cortex in both hemispheres: the right hemisphere (sky blue-colored tract)-no neural fibers reached the dorsolateral prefrontal cortex, orbitofrontal cortex, and posterior parietal cortex, and the left hemisphere (yellow-colored tract)-no neural fibers were connected to the orbitofrontal cortex) are noticed compared to a normal subject (73 year-old woman).


A 73 year-old female presented with anterior chest pain to the Emergency Department of our university hospital. She had chronic hypertension for 20 years and diabetes mellitus for 5 years. Her medications included aspirin 100 mg daily, clopidogrel 75 mg daily, ezetimibe 10 mg/rosuvastatin 20 mg daily, nifedipine 30 mg daily, telmisartan 40 mg, citagliptin phosphate 64.25 mg/Metformin 500 mg twice a day, and glimepiride 2 mg daily, but she did not take them regularly. On admission, blood pressure was 140/80 mmHg, pulse rate was 74 beats/min and she was fully conscious and did not show any neurologic deficits on physical examination. She was diagnosed with chronic stable angina pectoris, three-vessel disease, and chronic total occlusion of the left anterior descending artery by coronary angiography (CAG). Percutaneous coronary intervention was tried, but failed. Blood pressure, pulse rate, body temperature, and respiratory rate were stable, and she was transferred to the coronary care unit (CCU) for close observation. No life support such as oxygen supply, ventilator support, cardiopulmonary resuscitation, defibrillation, or left ventricular assist device was required, and the patient was able to intake foods orally. From the first day of CCU stay, she had been diagnosed with hyperactive delirium showing disorientation to time and place, restlessness, calling out, sleep disturbance, irritability, and refusal to cooperate with care, and quetiapine 12.5 mg was started and escalated to 25 mg twice a day. 5 days after CAG, she showed a deep drowsy mentality and could not obey a command and was seen by a neurologist. The motor function of the patient was checked to be Grade 4 or above in all extremities by the Medical Research Council (MRC) Scale for muscle strength, and the extraocular movement appeared normal. Brain magnetic resonance imaging taken on the same day revealed bilateral paramedian pontine infarction (Figure 1a). She was transferred to the Neurology Department and underwent conservative treatment; during this period, the neurological symptoms remained stable without any progression. 4 weeks after the onset of pontine infarction, she was transferred to the Department of Physical Medicine and Rehabilitation. She showed severe impairment of consciousness, with a Glasgow Coma Scale score of 7 (eye-opening: 2; best verbal response: 2; and best motor response: 3) and Coma Recovery Scale-Revised score of 10 (auditory function: 2; visual function: 3; motor function: 2; verbal function: 2; communication: 0; and arousal: 1) on the day of the transfer to our department. Her motor function was checked to be grade 4 in all extremity by MRC scale for muscle strength and could not eat orally because of drowsy mentality.

**Informed consent:** The patient’s daughter had provided signed, informed consent for publication of the patient’s information.
**Ethical approval:** The institutional review board of the university hospital approved the study protocol. The research related to human use has been complied with all the relevant national regulations, institutional policies, and in accordance with the tenets of the Helsinki Declaration, and it has been approved by the authors’ institutional review board or equivalent committee.

### 2.2 Methods

DTI was performed four weeks after the onset of pontine infarction using a 6-channel head coil on a 1.5 T Philips Gyroscan Intera with single-shot, spin echo-planar imaging. Imaging parameters were as follows: acquisition matrix = 96 × 96, reconstructed to matrix 192 × 192, field of view = 240 × 240 mm^2^, TR = 10.398 ms, TE = 72 ms, parallel imaging reduction factor = 2, EPI factor = 59, *b* = 1,000 s/mm^2^, and slice thickness = 2.5 mm. The Oxford Center for Functional Magnetic Resonance Imaging of the Brain Software Library (FSL v 5.0, www.fmrib.ox.ac.uk/fsl) was used to analyze DTI data (streamline samples: 5,000, step length: 0.5 mm, and curvature threshold: 0.2). Eddy current correction was applied to correct the head motion effect and image distortion. To reconstruct the ARAS, which plays an essential role in maintaining arousal and consciousness, the regions of interest were the pontine reticular formation at the level of trigeminal nerve entry zone and intralaminar thalamic nucleus at the level of the commissural plane for the lower dorsal ARAS [7], pontine reticular formation at the mid-pons level where the trigeminal nerve is seen and hypothalamus including the mammillary body, which was identified by the optic tract (anterior boundary) and mammillary body (posterior boundary) on the upper midbrain level for the lower ventral ARAS [8], and intralaminar thalamic nucleus at the level of inter-comissural plane between the anterior and posterior commissures to the cerebral cortex, namely, the medial prefrontal cortex (Brodmann area 32), dorsolateral prefrontal cortex (Brodmann area 8, 9, and 46), ventrolateral prefrontal cortex (Brodmann area 44, 45, and 47), orbitofrontal cortex (Brodmann area 10, 11, 12, and 47), premotor cortex (Brodmann area 6), primary mortor cortex (Broddman area 4), primary somatosensory cortex (Brodmann area 1, 2, and 3), and posterior parietal cortex (Brodmann area 5 and 7) for the upper ARAS [9]. Results of DTT for three portions of the ARAS in the patient were compared with those of a healthy subject, who was a 73 year-old woman with no history of the neurologic or psychologic disease. The neural connectivity in the left lower dorsal ARAS, both lower ventral ARAS, and both upper ARAS was impaired on DTT (Figure 1b).

## 3 Discussion

In the current study, three portions of the ARAS in a patient with impaired consciousness after bilateral pontine infarction were examined using DTT. Three portions of the ARAS were injured in both hemispheres: the lower dorsal ARAS – narrowing in the left side compared to the right and that of a normal subject, the lower ventral ARAS – non-reconstruction on both sides, and the upper ARAS – decreased neural connectivity to the right prefrontal cortex, right basal forebrain, and both basal ganglia and thalami. Impaired neural connectivity of the left lower dorsal ARAS, both lower ventral ARAS, and both upper ARAS revealed by DTT might be associated with impaired consciousness of the patient.

The ARAS mainly originates from the reticular formation of the brainstem and spreads to the cerebral cortex via projections through the thalamus and hypothalamus, which conveys impulses related to wakefulness and arousal [18]. Therefore, extensive damage of the ARAS following bilateral pontine infarction in our patient seems to have attributed to impairment of consciousness.

Elucidation of the exact brain structures that are responsible for the impairment of a patient who requires rehabilitation after brain injury is important because it can provide useful information about the extent of neural injury, potential for recovery, and possible targets for intervention (e.g. neurostimulation using transcranial direct current stimulation or repetitive transcranial magnetic stimulation) [19,20]. However, the ARAS cannot be clearly discriminated from adjacent structures using conventional brain MRI, functional neuroimaging techniques, electrophysiological methods, and MR spectroscopy because it contains many axonal fascicles projecting in different directions [21]. The recent development of DTT has enabled three-dimensional visualization and evaluation of the ARAS in the living brain.

Several previous studies have identified an association between the injured ARAS and impaired consciousness in different brain pathologies, including intracerebral hemorrhage, cerebral infarction in the bilateral thalamus and midbrain, traumatic brain injury, and hypoxic-ischemic brain injury [12,13,14,15,16,17]. However, no study has reported on the injury of the ARAS in bilateral pontine infarction. It has long been known that bilateral lesion in the pontine tegmentum can lead to the impairment of consciousness by hindering the function of the ARAS [21].

In conclusion, we demonstrated extensive disruption of the ARAS after bilateral pontine infarction using DTT, which seems to result in impaired consciousness in our patients. To the best of our knowledge, this is the first report to elucidate the injury of the ARAS in bilateral pontine infarction using DTT. However, because this is a single case report, further studies with a larger number of patients and follow-up data are needed to better understand the association between injury of the ARAS and impairment of consciousness, prognosis, and plan an appropriate rehabilitation strategy for this condition. In addition, further studies including other brain pathologies are needed to identify probable differences in the severity and/or prognosis of impaired consciousness based on the injured segments of the ARAS and pathogenic mechanism (e.g. cerebral infarction, intracerebral hemorrhage, traumatic brain injury, or hypoxic-ischemic brain injury).
